# Biogeochemical consequences of an oxygenated intrusion into an anoxic fjord

**DOI:** 10.1186/1467-4866-15-5

**Published:** 2014-04-28

**Authors:** Svetlana Pakhomova, Hans Fredrik Veiteberg Braaten, Evgeniy Yakushev, Jens Skei

**Affiliations:** 1 P.P.Shirshov Institute of Oceanology RAS, Nakhimovskii prosp. 36, Moscow 117991, Russia; 2 Norwegian Institute for Water Research (NIVA), Gaustadalléen 21, Oslo 0349, Norway; 3 Department of Chemistry, University of Oslo, Sem Sælands vei 26, Oslo 0371, Norway

**Keywords:** Anoxia, Oxygen intrusion, Hydrogen sulphide, Methylmercury, Iron, Manganese

## Abstract

**Background:**

This paper is based on the studies of the biogeochemical structure of the water column in the anoxic Fjord Hunnbunn (south-eastern Norway) performed in 2009, 2011 and 2012. This Fjord is an enclosed basin of brackish water separated by a narrow and shallow outlet to the sea with a permanently anoxic layer. We show how an oxygenated intrusion could lead to both positive and negative effects on the ecosystem state in Hunnbunn due to a change in the biogeochemical structure.

**Results:**

During the stratified periods in 2009 and 2012 the anoxic layer amounted to approximately 10% of the total water volume in the Fjord, while dissolved oxygen (DO) was present in 80-90% of the water. In the autumn of 2011 the water chemistry structure observed in Fjord Hunnbunn was clearly affected by a recent oxygenated intrusion defined by abnormal salinity patterns. This led to a shift of the DO boundary position to shallower depths, resulting in a thicker anoxic layer comprising approximately 40% of the total water volume, with DO present only in approximately 60% of the water. The oxygenated water intrusions led to a twofold decrease of the concentrations of hydrogen sulphide, ammonia, phosphate and silicate in the deep layers with a simultaneous increase of these nutrients and a decrease of the pH level in the surface layers. The concentrations of manganese, iron, and mercury species changed dramatically and in particular revealed a significant supply of iron and methylmercury to the water column.

**Conclusions:**

Oxic water intrusions into anoxic fjords could lead not only to the flushing of the bottom anoxia, but to a dispersal of sulphidic and low oxygen conditions to the larger bottom area. The elevation of the hydrogen sulphide to the shallower layers (that can be rapidly oxidized) is accompanied by the appearance in the subsurface water of methylmercury, which is easily accumulated by organisms and can be transported to the surrounding waters, affecting the ecosystem over a larger area.

## Background

Coastal marine systems, including estuaries, are not only important habitats for diverse marine animal life, but also a significant source of food for humans. Changing coastal marine ecosystems could therefore have a drastic impact on both animal and human life. Factors that are thought to stress the marine environment include de-oxygenation, acidification and ocean warming [1]. Oxygen depletion is shown to be a serious environmental issue in the oceans [2], in river-estuarine systems [3] and in coastal zones throughout the world [4,5]. Improving our understanding of the effects of the decrease of oxygen on biogeochemical processes and ecological state in the coastal areas is very important for evaluating risks and planning human activity there.

Since the 1960s, areas of hypoxia (less than 30% of oxygen saturation) in coastal zones have increased exponentially [4]. This is shown to be mainly caused by an increase of algae production in the surface waters due to eutrophication following increased inputs of nutrients from human activities. This resulting accumulation of organic matter in deeper waters and consumption of dissolved oxygen (DO) due to respiration, and high levels of stratification have led to the formation of hypoxic zones [4]. Studies suggest that hypoxia in the future will increase in extent, frequency and intensity in coastal areas [6].

Depletion of oxygen in coastal zones inevitably leads to biological impacts ranging from altered microbial activity (e.g. enhanced de-nitrification and sulphate reduction) to whole community displacements (e.g. loss of fisheries, invasion of displaced species into new habitats) [5]. Fluxes of contaminants, such as mercury (Hg), can vary due to redox changes, formation of metal sulphides and methylation of Hg under suboxic conditions [7]. This can significantly affect the water quality in connection with oxygen depletion. Decrease of oxygen and pH as well as the appearance in the water of the toxic and bioaccumulative form of Hg, methylmercury (MeHg [8]), may cause multiple stress impacts on biota. Hence, formation of hypoxia is a threat to healthy coastal ecosystems and thereby a direct danger to human health and economic welfare (i.e. fish kills) in coastal societies.

Mercury appears in the oxic surface waters of the oceans at low levels (sub ng/L), but can be elevated at the top of the food chain due to methylation of inorganic Hg into MeHg [9]. Even though marine seafood is considered the main source of human MeHg exposure [10,11] most research to date has focused on Hg methylation processes in freshwater systems. During oxygen depletion MeHg can be formed under suboxic and anoxic conditions in the sediments [12] and water column [7,13]. The fate of this MeHg, and its availability and exchange with surrounding waters, warrants further investigation.

Fjords and inshore waters are especially sensitive to the processes of hypoxia because of the restricted exchange with the sea [14]. Stratification due to the input of low density fresh water and topographic restrictions extends the residence time of bottom water and prevents aeration. In Norway, temporary anoxia occurs in areas of Oslofjorden [15] and permanent anoxia is found in Framvaren, Drammensfjord, Bærumsbassenget, Hunnbunn and Kyllaren [16-18].

Global change affects oxygenation events occurring during the cold periods of the year. Cold winters lead to an annual intensive oxygenation that prevents accumulation of large amounts of hydrogen sulphide (H_2_S) in the bottom layers. However, warm winters restrict the possibility of regular seasonal flushing, and H_2_S can accumulate in large concentrations. If a cold winter occurs after a series of warm winters, a “large scale” flushing can lead to the sudden appearance of large amount of H_2_S at the surface. Such events were observed in several Norwegian anoxic fjords in the winter of 2009/10 [19]. An extreme example of this is the flushing of the Fjord Framvaren which took place during the cold winter of 1941/42. A deep water renewal caused seawater with high levels of sulphide to be trapped underneath the ice cap of the fjord, leading to a complete fish kill [17].

The biogeochemical structure of redox zones in the water column of marine anoxic basins is characterized by an absence of overlap between DO and H_2_S and a presence of a so-called suboxic zone, formally defined as a layer where the concentration of both DO and H_2_S are below detection limits [20,21]. Under stable conditions, oxygen disappears at a depth where ammonia and dissolved manganese (II) are already observed, while H_2_S appears in deeper layers. In the redox zone reduced and oxidized forms of several elements (Nitrogen, N; Sulphur, S; Carbon, C; Manganese, Mn; Iron, Fe) can be observed, which reflects the complexity of processes occurring in this zone. As previously shown [22-25], Mn species have a dominating role in formation of the redox layer biogeochemical structure. The Mn species demonstrate clear transformation between different oxidation states, including Mn(II), Mn(III) and Mn(IV). The different states can form dissolved, particulate and colloidal compounds with an oxidizing capacity. Additionally, the distribution of manganese and iron species are a good indicator of changing redox conditions [23,24].

Processes occurring in changeable redox conditions can lead to dramatic consequences, i.e. the appearance of toxic H_2_S and alterations in the cycles of heavy metals, in particular leading to the formation of MeHg. In this study we examined the changes in the biogeochemical structure of the water column in the permanently anoxic fjord Hunnbunn during a period of water column stratification and after an intrusion connected with seasonal mixing. We have specifically focused on distributions of DO, H_2_S, and Mn, Fe, Hg species to study the effects on the fjord’s aquatic environment caused by a change in the hydrophysical patterns of the water column.

## Results and discussion

### Hydrophysical structure

**Figure 1:**
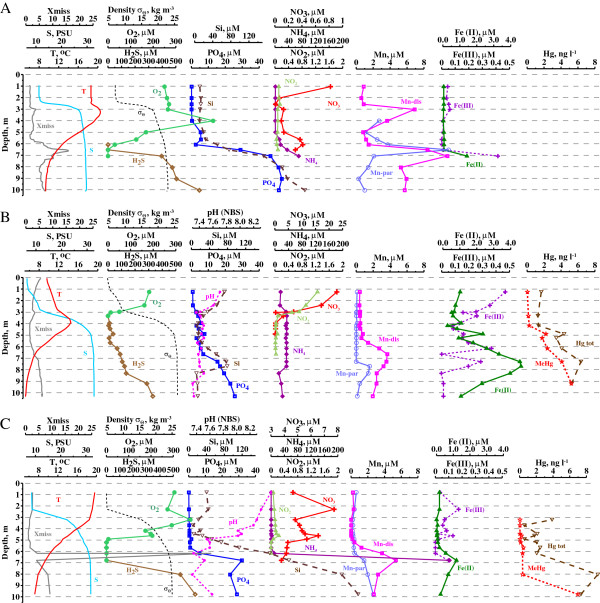
**Vertical distributions of temperature, salinity, turbidity density (σ**_**θ**_**), DO, H**_**2**_**S, phosphate, silicate, nitrate, nitrite, ammonia, dissolved Mn, particulate Mn, dissolved Fe(II), dissolved Fe(III), TotHg and MeHg in the Fjord Hunnbunn water column in August 2009 (A), October 2011 (B) and August 2012 (C).**

**Figure 2:**
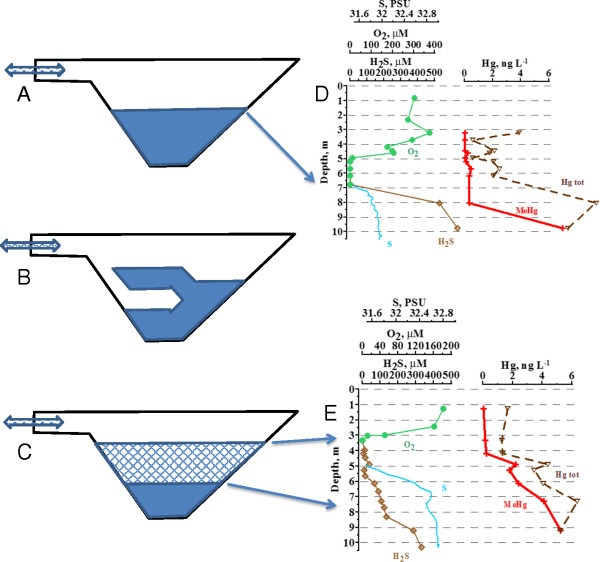
**A scheme of the sulphidic (solid blue fill), the oxic (white fill) and the low-sulphidic/suboxic (blue pattern fill) layers position the Fjord Hunnbunn before (A), during (B) and after (C) intrusion and observed vertical distributions of salinity, DO, H**_**2**_**S and Hg species in August 2012 (D) and October 2011 (E).**

The vertical structure of Hunnbunn showed a stable hydrophysical and biogeochemical state in 2009 (Figure 1A) and 2012 (Figure 1C), illustrated as the stratification period in Figure 2A. In 2011 the vertical structure showed unstable patterns (Figure 1B), illustrated as the mixing period in Figure 2C.

In both 2009 and 2012 the temperature profiles show warm (around 19.0°C) and unchanged temperatures in the top 3-4 m. Deeper than 4 m the temperature decreased in both years towards the minimum of 7-9°C (Figure 1A, C). In the autumn of 2011 (Figure 1B), profiles show temperature increasing from the surface (<10°C) to around 4 m depth (14.5°C) followed by a decrease towards the bottom (5°C).

In all the studied periods (2009, 2011 and 2012) there was a significant difference between the top layer of dilute brackish water (<10 PSU) and the bottom waters of higher salinity (approximately 30 PSU) with the halocline positioned at depths of 3 – 4 m (Figure A-C). The levels of salinity documented in the present study are similar to what is found in previous studies of the Hunnbunn fjord system [26].

**Figure 3:**
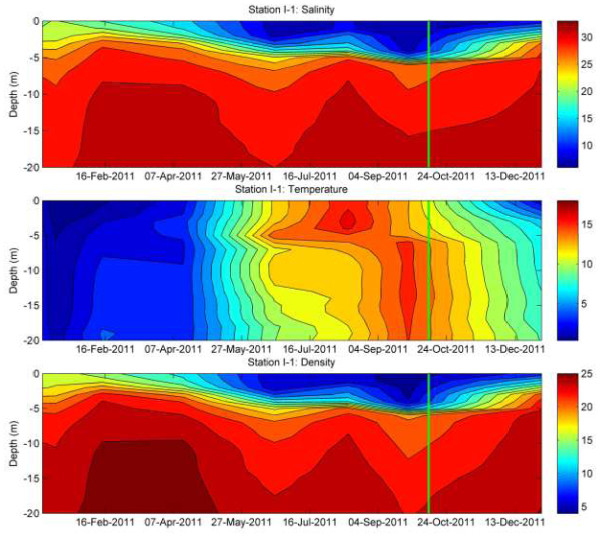
**Observed temporal variability of salinity (PSU; top), temperature, (°C; middle) and density, (kg/m**^**3**^**; bottom) in the water column at the monitoring station I-1 about 10 nm outside the Hunnbunn mouth (data from NIVA).** The green vertical lines mark the sampling date.

On a more detailed scale the salinity pattern (Figure 2E; inset) from 2011 reveals a well pronounced minimum (about 0.1 PSU) at the depth of 7.5 m. We hypothesize that this minimum of salinity can be explained by a low saline water intrusion at this depth, transported to the fjord from the surrounding waters. At a station I-1 positioned about 10 nautical miles outside the Fjord Hunnbunn mouth we observed a decrease of salinity with a minimum about two weeks before the sampling (Figure 3). Simultaneously, there was a decrease in temperature and an increase in density (Figure 3). This means that the water in the upper 2 m layer that was involved in an exchange with the Fjord Hunnbunn system became denser (the first time after the summer period, Figure 3) than the surface fjord’s water and should be transported into the deeper layers. This cold, low saline and oxygen rich water will affect the fjord’s biogeochemical structure.

The water column depth profiles from 2009 and 2012 (Figure 1A, C) demonstrate that the inflowing tide water does not penetrate into the deep layers of the Fjord waters. In these two profiles there are clear indications of a stable stratification (as shown by temperature, salinity, DO and H_2_S profiles). But when the tidal water gets cold and dense enough it penetrates into deeper waters (Figure 1B) and causes a mixing event.

The hydrophysical stability of the stratification periods in 2009 and 2012 is confirmed also by the turbidity profiles (Figure 1A, C). Both these years are characterised by the presence of turbidity maxima in the suboxic zone just above the appearance of H_2_S (6-7 m depth). This so-called Nepheloid Redox Layer (NRL) is observed in anoxic basins and caused mainly by particulate manganese and iron oxides and organic carbon compounds [23,27]. In 2011 the NRL was absent (Figure 1B).

### Dissolved oxygen and hydrogen sulfide

During the stratified periods (2009 and 2012) DO concentrations (Figure 1A, C) were high in the upper layers of the water column (0 – 5 m), but negligible at deeper levels (5 – 6 m). Both in May and August 2009 DO concentration reached more than 400 μM at a depth of 3-4 m, corresponding to a saturation value of about 200% [18]. In 2011, DO was only present in the upper 3 m of the water column (Figure 1C) where it never exceeded 200 μM.

In all three years of observations there were no overlap between DO and H_2_S (Figure 1A-C). A maximum thickness of the suboxic layer, a layer with both DO and H_2_S concentrations below the detection limits (i.e. 1 μM for DO and 0.3 μM for H_2_S), was 2 m in 2012. In 2009 and 2011 the suboxic layer was less than 30 cm, and in the neighbouring sample depths we observed either DO or H_2_S.

In 2009 and 2012 H_2_S was first detected at 6.5 and 7 m, and increased to 400 μM at 7 and 8 m respectively. The maximum concentrations were found in the bottom layer (10 m; 670 μM). In 2011 H_2_S was detected already at 4 m, and had uniform concentrations (not exceeded 40 μM) in the layer from 4 to 6.5 m. The near bottom maximum concentration reached 330 μM.

The oxygenated zone corresponded to 87, 59 and 82% of the total water volume of the Fjord Hunnbunn in 2009, 2011 and 2012 respectively (Table 1). The anoxic zones followed the opposite pattern and reached 13, 41 and 9% respectively. A significant suboxic zone amounted to 9% of total water volume only in 2012.

**Table 1:** Calculation results of elements content in total water volume of the Hunnbunn Fjord in studied years

**Parameter**	**2009**	**2011**	**2012**
Oxic zone, %	87	59	82
Anoxic zone, %	13	41	9
Suboxic zone, %	0	0	9
TotHg, g	-	31.1	31.6
MeHg, g	-	11.9	1.7
MeHg/TotHg > 20 %, %	-	40	<1

### pH and nutrient availability

pH was measured in 2011 (the mixing period) and in 2012 (stratified period). In 2012 pH was significantly different in the top layers compared to the deeper waters. From 1 – 4.5 m the pH slowly decreased from 8.5 to 7.9. Deeper than 5 m pH was always < 7.6. In 2011 pH levels were more stable throughout the water column, varying from 7.7 in the upper layer to 7.3 in the bottom waters.

During the stratified period in 2009 concentrations of phosphate, silicate and ammonia in the surface 0-5- m layer were low and did not exceed 0.3, 14 and 1.3 μM, respectively. From 5 m depth a sharp increase in their concentrations was observed. Phosphate, silicate and ammonia reached respectively 60, 250, and 400 μM at 10 m depth (Figure 1A). After the mixing event in 2011, the distributions of phosphate, silicate and ammonia were of irregular character in the upper layers (Figure 1B). In the upper 5 m layer their concentrations increased up to 20 times in comparison with 2009 and reached 6, 36 and 14 μM respectively. Deeper than 5 m their concentrations gradually increased to the bottom, but amounted to only half the values measured in 2009. In 2012 nutrient distributions were similar to those of 2009, but a sharp increase in concentrations started deeper, from approximately 7 m depth (Figure 1C). The maximum values of silicate and ammonia returned to the values before intrusion, while phosphate concentrations in deep layers were remained practically unchanged during the following year.

### Manganese and iron species distributions

In all three study years an increase of dissolved Mn concentrations started in the oxic zone (about 50 μM of oxygen) and its maximum (11, 3.7 and 5.4 μM in 2009, 2011 and 2012, respectively) was observed in the beginning of the sulphide zone at about 7 m, followed by its decrease to the bottom (6, 2.2 and 2.7 μM, respectively) (Figure 1). Increased dissolved Fe(II) concentrations were observed deeper than for dissolved Mn, at depths where oxygen was absent and H_2_S was present. Maximum observed Fe(II) concentrations at 7 m reached 1.4, 4.6 and 1.2 μM in 2009, 2011 and 2012, respectively. In 2011 (after the mixing) dissolved Fe species distribution showed, as for nutrients, irregular character from the surface to 5 m depth with high concentrations of up to 0.5 and 2.4 μM for Fe(III) and Fe(II), respectively (Figure 1B).

In 2009, a maximum of particulate Mn of 11 μM was positioned at the redox interface, just above the dissolved Mn maximum (Figure 1A). In 2011 it decreased 10 times and moved deeper, below the dissolved Mn maximum. In 2012 concentrations of particulate Mn gradually increased from 5 m depth to the bottom (Figure 1C).

The Mn species distributions at the redox interface in the Hunnbunn in 2009 were typical to those in other anoxic basins in the stable state.

The same character of irregularities in Fe and Mn species distribution as in the Hunnbunn in 2011 has been observed in the redox zones of the Black and Baltic Seas following oxygen intrusions into the anoxic zone [23,28,29]. If these intrusions led to total disappearance of H_2_S, dissolved Fe(II) also disappeared at this depth, dissolved Mn(II) decreased to small values (sometimes below the detection limit), and a maxima of particulate Mn(IV) and dissolved Mn(III) were formed. Over a short period after the intrusion the distributions of DO, H_2_S and Fe return to their initial states, but the distribution of Mn species has an irregular character for a longer time [23].

In the case of the Fjord Hunnbunn the behaviour of Mn species is typical for an intrusion event, i.e.a dissapearence in 2011 of particulate Mn at the redox interface, formation of particulate Mn maximum at the intrusion depth with simultaneous decreas of Mn(II). Absence of particulate Mn maxima in the redox interface in 2012 indicate that Mn species distribution did not returned yet to the equilibrium state.

Distribution of dissolved Fe(II) and Fe(III) in 2009 and 2012 are similar to those observed in stable redox zones in other sites [23-25,29]. An increase of Fe(II) concentration after intrusion is most likely caused by dissolution of particulate iron sulphides under twofold decrease of H_2_S concentration. When the concentration of H_2_S returned to the level observed before intrusion, the concentration of dissolved Fe(II) decreased again. This is closed to observed previously in Norwegian fjords and correspond thermodynamical calculations.

### Mercury processes

The profile measurements from 2011 showed that TotHg concentrations varied between 1.3 and 1.7 ng/L from surface down to 4 m (Figure 1B, 2E). Then concentrations increased to 3.4 - 4.4 ng/L at 5 m, and the maximum concentration of 6.4 ng/L were found at 7 m depth. MeHg concentrations increased regularly from the surface layer (0.04 ng/L) to the bottom (5.22 ng/L), corresponding to 2.1 - 99.4% of TotHg (Figure 1B).

In 2012 the TotHg concentrations showed a similar pattern as in 2011 between 3.5 and 6 m depth, with concentrations increasing from 0.6 to 2.5 ng/L. The maximum of 9.4 ng/L were found at 8 m depth (Figure 1C, 2D). In 2012 TotHg levels were also relatively high at 3 m depth (3.9 ng/L) compared to 2011 (1.3 ng/L). MeHg concentrations in 2012 were generally lower than in 2011 between 3 and 8 m depth (<0.5 ng/L), but an increase occurred at 10 m (7.0 ng/L) where MeHg constituted 95% of TotHg.

The levels of TotHg are similar to that found in other water basins with no local mercury input [30] but lower than in contaminated sites [31]. Our upper layer water measurements of MeHg revealed concentrations similar to what is found in oxic surface waters [32,33]. However, the concentrations of MeHg found in deeper layers were significantly higher than what is observed even in contaminated sites [31]. MeHg/TotHg ratios in water column could vary up to about 10% in maxima [13,30] and never was found so high that those were observed in Hunnbunn in 2011.

The highest concentrations of MeHg (and MeHg/TotHg ratios) were found in the deepest layers of the Hunnbunn in both periods (stratified and after mixing), that testify to MeHg production in the sediments [12,34]. Meanwhile in some other sites the highest MeHg/TotHg ratio is found in the top of the suboxic zone, where DO and sulphide are low, circumstantially suggesting that production of MeHg is controlled by sulphide speciation [7,30] or in other sites MeHg is produced in the sediments [12,34]. Since we did not find maximum MeHg concentrations in the suboxic layer, but rather found MeHg concentrations to increase toward the sediments, we conclude that the main MeHg source in Hunnbunn is likely the sediments rather than the water column. In addition to sediment production, the water column profile of 2012 could also indicate that the bottom water acts as a sink for MeHg. Injection of MeHg along with the oxygenated intruding water coincides with the profile observed in 2011. Following this hypothesis, the profile in 2012 suggests that the anoxic bottom waters are a sink for MeHg, because concentrations of MeHg increase towards the bottom (Figure 1C).

The distribution of the Hg species in 2012 approximates those observed in marine environment [13,30], and we suppose that these are characteristic for the Hunnbunn stable state and those for 2011 are coused by intrusion event.

In 2011, 31.1 g of TotHg were present in the whole water mass of the Hunnbunn. This changed very little the next year; in 2012, 31.6 g of TotHg were present. For MeHg the situation is different. In 2011, 11.9 g of MeHg was present in the fjord Hunnbunn (approximately 38% of the total). In 2012 however, only 1.7 g of MeHg was present (approximately 5%). In the stratified period (2012) MeHg/TotHg ratios exceeding 20% are present only below 10 m depth, or in less than 1% of the total water masses. After mixing, MeHg/TotHg ratios higher than 20% were present already at 4.3 m depth, corresponding to approximately 40% of the water masses.

The above calculations of integrated concentrations of TotHg and MeHg show that the amount of TotHg in the fjord is relatively stable over time. This indicates either that small amounts of Hg species are transported into and out of the fjord, or that the flux out of the fjord is equalized by the input sources around the fjord.

The amount of MeHg in the whole water basin was substantially higher in 2011 than in 2012 (Table 1). This circumstantially suggests that more MeHg is in the basin when an intrusion happens.

The increased concentrations of MeHg in the fjords water in 2011 can be explained by the following mechanisms: 1) MeHg was injected with the intrusion waters 2) MeHg is transported to the water column via re-suspension of sediments during flushing/mixing [35,36]. 3) MeHg diffuses into the water column from a larger area of the anoxic bottom, where MeHg was formed after the intrusion due to sulphate reduction and dissolution of Fe and Mn oxyhydroxides, and formation of sulphides [31,37,38], or 4) MeHg was formed in the water column under low hydrogen sulphide (or suboxic) conditions as a result of the intrusion.

We suppose that the mechanism (1) is not relevant, because, it is unlikely that the oxygenated water intruding into the fjord had MeHg concentrations as high as what we observed in the profile (approximately 2 ng/L). The water intruding into the fjord is evidently oxic and must be considered surface water. Surface waters of Norwegian fjords (and open sea water) do not have concentrations of MeHg this high.Mechanism (2) is not relevant either, because the bottom sediment re-suspension under anoxic conditions should lead to an increase in the concentrations of Mn, Fe and phosphorous in the bottom layer [39]. But in 2011 we observed a decrease of Mn, Fe and phosphorous concentrations toward the bottom layers (Figure 1B).

More likely our observations correspond to findings of [37]. The sediments, especially during anoxic events, should be considered as a primary source of MeHg for the water column supporting mechanism (3). We do not know the MeHg concentrations in the pore water or in the sediments, but if the concentrations in the pore water are elevated, diffusive transport of MeHg to the bottom water is likely [38].

The formation of MeHg in the water column at low hydrogen sulphide (or suboxic) conditions as a result of the intrusion (mechanism (4)) is also possible. However, previous studies have shown that the main production of MeHg (maximum MeHg concentrations) occur at the suboxic interface [13,30]. Due to our maximum concentrations towards the bottom layers, we do think this is less likely than mechanism (3).

Nevertheless in any case we obviously observe an increase of MeHg caused by an intrusion. This larger amount of MeHg affects a larger amount of water, with potentially severe effects on the ecosystem in Hunnbunn.

The MeHg concentrations found in the bottom waters of enclosed, or partly enclosed basins will depend on the residence time of the water (i.e. how often one has intrusions). Following this argument, the accumulated storage of MeHg in the bottom layers will be emptied during a renewal following an intrusion. After the intrusion, the accumulation of MeHg will continue. The environmental consequence is that the surface waters (and species living there; e.g. plankton, fish and shellfish) are exposed to increased concentrations of MeHg. This might occur in the Hunnbunn basin as well as outside the basin (i.e. the surrounding sea water). Following the hypothesis discussed in [37], the reversion to oxic conditions removes Hg species from the water column, most probably through precipitation and coprecipitation with Fe and Mn oxyhydroxides that leads to a decrease of MeHg/TotHg ratio in the water column decreases from 100% to nearly 0. It is conceivable that some demethylation process is occurring in the water column and that Fe/Mn oxyhydroxides scavenging of MeHg is more efficient than on Hg.

### Consequences of the intrusion

In 2009 the distributions of all studied chemical parameters in the Hunnbunn were typical of those in other anoxic basins [21,24,25]. This leads to the conclusion that the Hunnbunn biogeochemical system was in a stable state at that time. In 2011 the distributions of chemical elements together with patterns of salinity abnormality indicated that an oxygenated water intrusion into the anoxic layer (at 7.5 m depth) took place sometime prior to sampling. The short term consequences of this intrusion are:

1. Shift of H_2_S boundary to a shallower depth that leads to a decrease of the oxic zone and an increase of the anoxic zone by about 30% of fjord water volume and dispersal of anoxia to the larger part of the bottom. For a small fjord like Hunnbunn this is a significant change which diminishes the water volumes suitable as a biological habitat with respect to pelagic life and bottom fauna.

2. Concentrations of all the nutrients in the subsurface layer (3-5 m) increased by an order of magnitude and simultaneously the pH level decreased. Together these effects could lead to changes in the ecosystem state of the Fjord with shift of biological species and potentially enhance of primary production.

3. Below the redox zone (below 5 m depth) concentrations of H_2_S and all nutrients decreased by a factor of two.

4. MeHg absolute mass in the Fjord’s water volume increased up to an order of magnitude after the intrusion. Potentially, this form of Hg appeared in water depths shallow enough to be transported with currents from the fjord to surrounding waters. Whether this transport occurred or not, the availability of MeHg increased significantly.

By about one year after the intrusion, the volume of the oxic zone in the fjord had returned to the level prior to the intrusion, while the H_2_S water volume had become even lower. The distribution of Fe, silicate and ammonia returned to initial (equilibrium) state but this did not happen for Mn and phosphate. It has been shown earlier [40] that different times are needed for redox processes for different elements. Besides, it depend on the bacterial community, since the reactions in natural waters are commonly mediated by bacteria. For the sea water (the Black Sea) these reactions are very quick for Fe and much slower for Mn [22,24]. Modelling experiments also showed that more time is needed for Mn species to return to initial stable state after intrusion (as much as two years compared to sulphide for the Gotland Deep conditions, [41]). MeHg concentration in water column decreased significantly next year together with redox conditions re-establishment. It seems that both oxic and high anoxic waters are a sink for MeHg and it could exist in very narrow redox conditions and could be demethylazed enough fast. The fate of the stability of MeHg in different water masses and conditions should be carefully studied.

A sporadic or regular significant temporal decrease of the oxic zone volume and change of nutrient regime and pH level could result in a serious change of the ecosystem state. Historical data from Hunnbunn reveal that the oxygen conditions there were much better in the past. This is based on observations of oysters in the 19th century [26]. An intensive eutrophication period and an absence of periodic dredging after 1950s already led to changes in the surface layer ecosystem: disappearance of the oysters, decline of sea grass beds and the appearance of large amounts of green algae [26]. The latter results in formation of a water layer with very high oxygen concentration during the bloom period. Extremely high super-saturation of oxygen at 2.5 m depth was measured in May 2009, with up to 250% super-saturation; the same phenomenon was also observed in August 2009 [18]. Equally large super-saturation of oxygen was previously observed in June 1999, but that year the super-saturation had disappeared about a month later [26,18]. Thus, the measurements in 2009 show a more enduring high primary production than in 1999, and this may indicate that the water quality has deteriorated over the last 10 years.

The long term consequences of an intrusion could be that the volume of anoxic water and content of H_2_S in the water mass of the fjord decreases. Extent and duration of this decrease depend on the intrusion intensity. In the Hunnbunn it seems that the system recovers relatively quickly because of high concentration of nutrients, metals and organic matter in the sediment that probably lead to substantial benthic fluxes.

The most important long term consequence of oxic intrusion into the anoxic zone of Hunnbunn is the appearance of MeHg in a larger volume of the fjord water, including the pycnocline. MeHg is easily bioaccumulated by organisms which can be caught here or transported to the surrounding waters, thereby affecting the ecosystem state over a larger area and also human health.

## Experimental

### Study area

**Figure 4:**
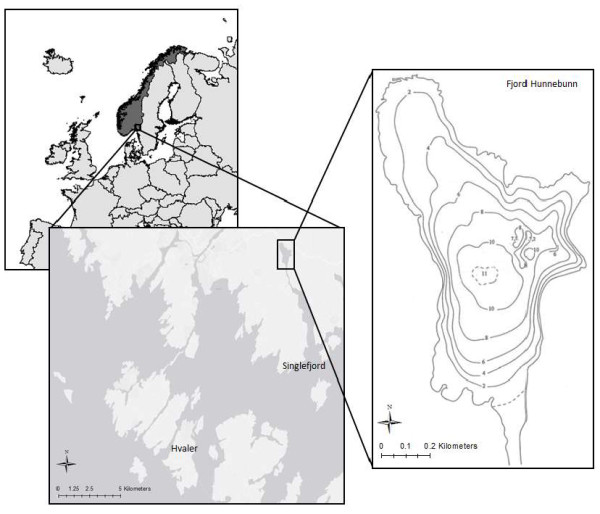
**Location and bathymetry map of the Fjord Hunnbunn, modified from [**[18]**].** The contours in the bathymetry map refer to the depth in meters.

Field studies were performed in the fjord Hunnbunn situated in south east Norway (Figure 4; Table 2). Hunnbunn is a small (0.99 km^2^) enclosed fjord connected to more open water (Singlefjord) through Thalbergsund, a long, narrow and shallow channel (1.8 km long; 30 - 125 m wide; 1 – 1.5 m deep). The fjord basin has an average depth of 5.2 m, with a maximum depth of 11 m. The total volume of water is 5150000 m^3^ (Table 3). The catchment area (11 km^2^) consists of mainly agriculture land (>90%) [18].

**Table 2:** General characteristics of the fjord Hunnbunn

**Characteristics**	**Unit**	**Specification**
Position	UTM; (x, y)	(275740.9, 6570035.7)
Fjord area	km^2^	0.985
Catchment area	km^2^	~ 11
Average depth	m	5.2
Max depth	m	11.0
Coastal length	km	6.5

**Table 3:** The total area and volume of water (absolute and relative) per depth metre in the Hunnbunn estuary

**Depth (m)**	**Area (km**^**2**^**)**	**Volume (10**^**4**^ **m**^**3**^**)**	**Volume (%)**
0	0.99	515	100.0
2	0.77	337	65.4
4	0.57	203	39.4
6	0.39	107	20.8
8	0.25	43	8.3
10	0.09	10	1.9
11	< 0.01	< 1	< 0.1

The fjord has only two small stream inlets. In combination with the narrow outlet these features make Hunnbunn a relatively isolated and permanently anoxic estuary. During transport through this narrow outlet (a 5 km long channel with 2 m minimum depth and tidal velocity up to 100 cm/sec), the incoming water homogenises and oxygenates the water. Previous studies reveal how the surface water in Hunnbunn is permanently brackish (<10 PSU) and bottom water salinity never exceeded 25 PSU throughout the year 1999 [26]. Despite the shallow connection with Singlefjord, the horizontal currents through the channel are significant. The tide water amplitude is up to 45 cm and the currents can be as strong as 15 - 33 m^3^/s (tidal pump). The effect of freshwater input from streams (yearly average < 0.1 m^3^/s) on the water composition in Hunnbunn is therefore low compared to the tidal pump effect [18].

### Sampling

Distributions of chemical parameters in Hunnbunn were studied in May and August 2009, October 2011 and August 2012. The sampling point was positioned at the deepest part of Hunnbunn (Figure 4). Water samples for chemical measurements were collected using pump systems. We used an on-board 12 V peristaltic pump, with an 11 mm hose attached to the CTD (conductivity-temperature-depth) probe on board a small boat. The time of the hose flushing was about 1 min for the 11-m hose used in the fjord. These pump systems allowed us to sample water protected from atmospheric oxygen. This is essential to avoid oxygen contamination in the suboxic water samples. In our studies we used the AANDERAA Optode 3835 sensor to measure oxygen concentrations in the hose during the sampling. Sampling in the suboxic layer was performed at 0.15 – 1 m intervals, aiming to obtain closely spaced data for the distribution of the measured chemical parameters.

### Chemical analysis

DO (Winkler), H_2_S, phosphate, silicate, nitrate, nitrite, ammonia, dissolved Mn, Fe(II) and Fe(III) were measured using standard methods described in [42,43]. Water samples for Fe and Mn species analysis were collected directly from hose to the plastic tubes for analysis both for total concentration (unfiltered samples) and for dissolved forms (filtered using a syringes Millipore 0.4 μm filters). Photometric determination of metals concentration was conduct in 2-4 hours after sampling. Concentration of particulate manganese was calculated by difference between unfiltered and filtered samples. Precision of dissolved metal analysis was typically 3%. Detection limits were 20 and 100 nM for iron and manganese, respectively [22,43].

pH (NBS scale) was measured with a pH-meter (Metrohm 780).

Water samples for Hg species analysis were collected in 250 ml fluorinated polyethylene (FLPE) bottles. Total Hg (TotHg) and MeHg were sampled in individual bottles to avoid errors caused by loss of Hg during preservation [44]. The analysis method for MeHg is based on USEPA Method 1630 [45] for determining MeHg in water by distillation, aqueous ethylation, purge and trap, and cold vapor atomic fluorescence spectrometry (CVAFS). The method for TotHg is based on USEPA Method 1631 for determining Hg in water by oxidation, purge and trap and CVAFS [46]. The method detection limit is 0.02 ng/L and 0.1 ng/L (3 standard deviations of method blanks) for MeHg and TotHg, respectively. For both species automated systems were used for analysis (Brooks Rand Labs MERX automated systems with Model III AFS Detector). Due to low concentrations of particulate matter all samples were analysed unfiltered.

### Total mercury concentration calculations

By comparing the values from Figure 4 (concentration of TotHg and MeHg at different depths) and Table 3 (volume of water at different depths) we can calculate the approximately total amount of TotHg and MeHg present in the Hunnbunn water basin in 2011 and 2012. The calculations are based on a simple integration by choosing a concentration that represents the different depth areas in Table 2 (Because of the small water volume deeper than 11 m we have chosen to disregard this depth). In areas were more than one concentration value is available we used the mean concentration. This concentration is then multiplied with the total volume of water to obtain an estimation of the total amount of TotHg and MeHg at different depths and in the total water mass.

Since we tried to capture the transition from oxic to anoxic waters in as much detail as possible, not all depth areas are present with concentrations of the Hg species. For example, no measurement was done in the 8-10 m depth area in 2011 or in the 0-2 depth area in 2012. However, we have in these two situations done the following: To represent the concentrations of TotHg and MeHg in the 8-10 m depth area in 2011 we used the mean concentrations of the 6.5 and 10.5 m depth measurements and we used half the concentrations in the 2-4 m depth area to represent the 0-2 m depth area in 2012.

## Conclusions

Flushing events lead to positive and negative effects on the ecosystem state of the anoxic fjord Hunnbunn. The short time consequences are negative because larger water volume and bottom area became anoxic immediately after intrusion. In addition, large amounts of nutrients appear in the subsurface layer in combination with a significant decrease of pH. This could possibly lead to changes in the ecosystem state of the fjord with a shift of biological species and enhanced primary production. In the case of Hunnbunn, a disappearance of oysters has been documented [26]. A positive long term consequence is that H_2_S and nutrient levels in the water mass decrease for a while after intrusion. Periodic flushing may therefore improve the state of the fjord ecosystem.

The most important negative consequence, both short and long term, is that sporadic or regular flushing events in anoxic fjords like this lead to an appearance in the subsurface (quite shallow) water of MeHg. This compound is easily accumulated by organisms like fish and can be transported to the surrounding waters, thus affectingthe level of mercury in fish over a larger area. We hypothesize that during warm winters, coastal anoxic basins such as fjord Hunnbunn accumulate large amounts of H_2_S and MeHg, while during cold winters, flushing events transport mercury to the surrounding waters. The fate of the stability of MeHg in different water masses and conditions should be carefully studied.

## Competing interests

The authors declare that they have no competing interests.

## Authors’ contributions

SP carried out sample collection, manganese and iron analyses, interpreted the results and drafted the manuscript. HFVB carried out mercury analyses and helped to draft the manuscript. EY carried out sample collection, inorganic geochemistry analyses (DO, H_2_S, pH, etc.) and helped to draft the manuscript. JS participated in the design of the study, sample collection and helped to draft the manuscript. All authors read and approved the final manuscript.
